# Association between Vaginal Infections and the Types and Viral Loads of Human Papillomavirus: A Clinical Study Based on 4,449 Cases of Gynecologic Outpatients

**DOI:** 10.1155/2020/9172908

**Published:** 2020-03-24

**Authors:** Wei Wang, Xian-hui Zhang, Mei Li, Chong-hua Hao, Hong-ping Liang

**Affiliations:** ^1^Department of Laboratory Medicine, Shanxi Provincial People's Hospital, Taiyuan, China; ^2^Department of Laboratory Medicine, Children's Hospital of Shanxi Province, Taiyuan, China; ^3^Department of Gynecology, Taiyuan People's Hospital, Taiyuan, China

## Abstract

**Objective:**

We here evaluated the association between human papillomavirus (HPV) infection and vaginal infections, including bacterial vaginosis (BV), trichomonas vaginalis (TV), and vulvovaginal candidiasis (VVC).

**Methods:**

A total of 4,449 women were enrolled in this study and given gynecological examinations. HPV genotyping and viral load determination were performed using a real-time PCR. Vaginal infections were diagnosed using wet mounts of vaginal secretions, gram-stained vaginal secretion smears, and chemical enzyme kits.

**Results:**

In this study, the overall HPV-positive rate was 25.06%, and vaginal infection tended to occur in women with HPV infection (*P* < 0.05). HPV infection tended to occur in BV- and TV-positive women (*P* < 0.05) and not in women with microecological disorders, intermediate type BV, VVC, or coinfection (*P* > 0.05). The most common genotypes were HPV58 and HPV53 in women with normal vaginal microecology and HPV16 and HPV52 in the women suffering from vaginal infection. The viral loads among groups for HPV16 and HPV52 showed no statistically significant differences (*P*=0.940; *P*=0.167).

**Conclusions:**

Our study revealed that BV and TV are associated with HPV infection, especially high-risk HPV infection, while VVC has no association with HPV infection. Further studies are needed to explore the detailed mechanism.

## 1. Introduction

Cervical cancer is one of the leading causes of death from gynecologic malignancy, and it has an estimated 530,000 new cases annually worldwide with 270,000 deaths [1]. In China, about 106,430 new cases of cervical cancer are diagnosed annually, with 47,739 deaths (estimates for 2018). Cervical cancer is the sixth major cause of female cancer and the eighth major cause of female cancer deaths in China [2]. Most cases of cervical intraepithelial neoplasia (CIN) and cervical cancer are caused by oncogenic human papillomavirus (HPV) infection, with HPV infection identified in approximately 95% of invasive cervical cancer [3, 4]. For most women, HPV infections are transient and spontaneously cleared by the host with no intervention or clinical consequences. However, 5%–10% of HPV infections cannot be cleared, and they become persistent infections that can cause cervical and other types of cancer. It is difficult to predict whether HPV infection would disappear spontaneously or lead to malignant transformation.

Various factors are thought to increase the likelihood of the persistent infection and subsequent tumor formation. These include age, high parity, oral contraceptive use, cigarette smoking, lesion grade, HPV genotype, viral load, and coinfection with human immunodeficiency virus [1, 5, 6]. Some previous studies have shown that the vaginal infections could disrupt the innate defenses of the balanced vaginal ecosystem and affect the immune competence for clearance of HPV infection [7–9]. Bacterial vaginosis (BV), trichomonas vaginalis (TV), and vulvovaginal candidiasis (VVC) are the most common vaginal infections. They are associated with high levels of anaerobes. The anaerobes and their metabolites may degrade cervical mucus, destroy vaginal epithelial cells, and break the innate defenses of the vaginal environment. Despite similar effects of vaginal infections, their role as risk markers for HPV infection remains controversial. In addition, there are relatively few studies that analyze the relationship of the HPV genotype and viral loads in these vaginal infections. The purpose of the current study was to evaluate the association between vaginal infections and the types and viral loads of HPV in a large cohort of gynecologic outpatients.

## 2. Materials and Methods

### 2.1. Study Subjects

From July 2018 to May 2019, a total of 4,449 women (aged 18–86 with a mean of 42.8 ± 11.1 years) were included in the study at Shanxi Provincial People's Hospital. All subjects were given gynecological examinations, and cervical and vaginal specimens were collected. The study was approved by the ethical committee of Shanxi Provincial People's Hospital.

Wet mounts of vaginal secretions were used to detect *Trichomonas* in vaginal secretions. The vaginal swab samples were rolled onto slides for Gram staining, and the remaining samples were used to detect indicators of inflammation and microbial function. Gram-stained vaginal secretion smears were used for laboratory tests of hyphae and spores of *Candida*, vaginal cleanliness, leukocytes, and clue cells. The diagnosis of BV was based on Gram staining and Nugent's scoring system [10]. A chemical enzyme kit (Shuoshi Biotechnology Co., Ltd., Jiangsu, China) was used to detect indicators of inflammation and microbial function, including hydrogen peroxide, leukocyte esterase, sialidases, beta-glucuronidase, acetyl glucosaminidase, and pH. Abnormalities in any of the following indicators can be diagnosed as microecological disorders, including concentration and diversity of bacteria and inflammatory indicators such as leukocyte count of vaginal secretion, pH value, and lactobacillus function [11].

### 2.2. HPV Genotyping and Determination of Viral Load

Cervical secretions were collected by physicians with endocervical cotton swabs and used for HPV testing. A real-time PCR assay (Shuoshi Biotechnology Co., Ltd., Jiangsu, China) was used to detect HPV infection [12]. PCR probes and primers were designed for 21 HPV genotypes, including 18 high-risk types (HPV-16, 18, 26, 31, 33, 35, 39, 45, 51, 52, 53, 56, 58, 59, 66, 68, 73, and 82) and three low-risk types (HPV-6, 11, and 81) that are common in China [2]. To account for the discrepancy in the number of cervical epithelial cells collected in the specimen, levels of DNA copies of input cervical epithelial cells were also determined. The normalized viral loads of HPV were expressed as copies/10^4^ cervical epithelial cells.

### 2.3. Statistical Analyses

The data concerning HPV prevalence were analyzed by the binary logistic regression method and chi-squared analysis. The viral load of HPV was log_10_ transformed prior to analysis, and the differences among groups were analyzed by Kruskal–Wallis *H* test. The SPSS software version 22.0 (IBM Company, Chicago, IL, USA) was used to analyze all data, and two-sided *P* values under 0.05 were considered statistically significant.

## 3. Results

### 3.1. HPV Infection and Vaginal Infections

A total of 1,115 (aged 18–85 with a mean of 43.6 ± 11.2 years) of 4,449 (25.06%) women in this study were found positive for HPV, with 16.66% (741/4,449) infected with a high-risk single type, 1.21% (54/4,449) infected with a low-risk single type, and 7.19% (320/4,449) infected with multiple types. Among the 4,449 women, there were 11.80% (525/4,449) BV infection, 28.43% (1,265/4,449) intermediate type BV, 38.37% (1,707/4,449) microecological disorders, 11.35% (505/4,449) normal vaginal microecology, 5.53% (246/4,449) VVC, 1.82% (81/4,449) TV, and 2.70% (120/4,449) coinfection (Table 1).

Among the 1,115 women with HPV infection, 24.48% (273/1,115) suffered from vaginal infection (BV, VVC, TV, and coinfection), while 20.97% of the 3,334 (699/3,334) women without HPV infection suffered from vaginal infection. There was a significant difference in the prevalence of vaginal infection between the women with and without HPV infection (*χ*^2^ = 6.058, *P* < 0.05), and the prevalence in HPV infected women was higher.

We compared the rate of HPV infection in each group with that of the normal vaginal microecology group. There was a significant difference between women with BV infection, TV infection, and normal vaginal microecology (*P* < 0.05), while there was no significant difference between women with microecological disorders, intermediate type BV, VVC, coinfection, and those with normal vaginal microecology (*P* > 0.05). When the OR value of the normal vaginal microecological group was 1, the vaginal infection and risk of HPV infection were analyzed. There was a positive correlation between BV, TV infection, and HPV infection (*P* < 0.05), but there was no correlation between microecological disorders, intermediate type BV, VVC or coinfection, and HPV infection (*P* > 0.05). The results showed that BV and TV infection may be risk factors for HPV infection (Table 2).

### 3.2. HPV Types and Vaginal Infections

The frequency of type-specific HPV infection among groups was observed via real-time PCR assay. The most common HPV genotypes in the normal vaginal microecology group were HPV58 and HPV53 (accounting for 1.98%). The most common HPV genotypes in the microecological disorder group, intermediate type BV group, BV group, VVC group, TV group, and coinfection group were HPV16 (accounting for 3.34%, 3.87%, 6.48%, 2.85%, 3.70%, and 4.17%, respectively) followed by HPV52 (accounting for 2.64%, 2.61%, 3.24%, 2.03%, 2.47%, and 4.17%, respectively) (Table 3).

The prevalence of a single high-risk HPV genotype in the normal vaginal microecology group, microecological disorder group, intermediate type BV group, BV group, VVC group, TV group, and coinfection group was 17.43%, 17.46%, 16.36%, 23.05%, 15.45%, 24.69%, and 19.17%, respectively, and there was significant difference among the seven groups (*χ*^2^ = 18.052, *P* < 0.05). The prevalence of a single low-risk HPV genotype in the seven groups was 1.39%, 1.41%, 0.79%, 0.76%, 2.44%, 2.47%, and 0.83%, respectively, and the prevalence of multiple HPV genotypes was 5.94%, 6.85%, 8.06%, 8.38%, 4.47%, 9.88%, and 6.67%, respectively. There were no significant differences between these two infection genotypes among the seven groups (*χ*^2^ = 7.725, *P* < 0.05; *χ*^2^ = 7.678, *P* < 0.05) (Table 3).

### 3.3. HPV Viral Load and Vaginal Infections

**Figure 1:**
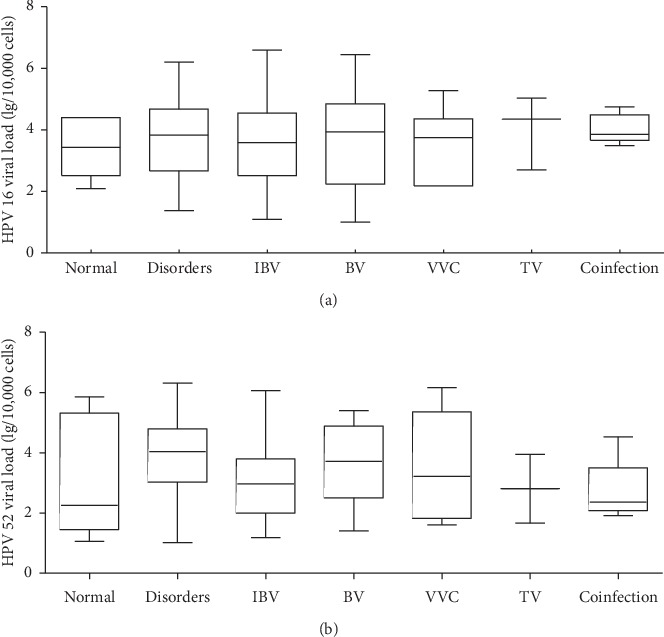
HPV16 and HPV52 viral load according to the groups. There were no statistically significant differences among the groups for HPV16 (a) and HPV 52 (b) (*H* = 1.763, *P*=0.940; *H* = 9.123, *P*=0.167). Normal, normal vaginal microecology group; disorders, microecological disorder group; IBV, intermediate type BV group; BV, bacterial vaginosis group; VVC, vulvovaginal candidiasis group; TV, trichomonas vaginalis group; coinfection, coinfection group.

We analyzed the viral load (log10/10,000 cells) of HPV16 and HPV52 among groups. The HPV16 viral loads of the normal vaginal microecology group, microecological disorder group, intermediate type BV group, BV group, VVC group, TV group, and coinfection group were 3.44 (2.52–4.40), 3.83 (2.67–4.68), 3.59 (2.52–4.55), 3.94 (2.24–4.85), 3.75 (2.18–4.36), 4.35 (2.70–), and 3.86 (3.67–4.49), respectively. The HPV52 viral loads of seven groups were 2.26 (1.46–5.32), 4.04 (3.03–4.80), 2.97 (2.00–3.80), 3.71 (2.51–4.88), 3.22 (1.83–5.36), 2.81 (1.67–), and 2.36 (2.08–3.50), respectively. The viral load comparisons among groups for HPV16 and HPV52 showed no statistically significant differences (*H* = 1.763, *P* = 0.940; *H* = 9.123, *P* = 0.167, respectively; Figure 1).

## 4. Discussion

Infection with high-risk HPV is a precondition for CIN or cervical cancer [1–3]. HPV infection is associated with a number of factors, including age, oral contraceptive use, pregnancy, and impaired immune function [5, 6]. However, there are usually no obvious clinical signs or symptoms after HPV infection, which results in difficultly in assessing the risk factors for persistent HPV infection. Recent studies have shown that, in addition to the aforementioned factors, vaginal microecological changes may be associated with increased odds for HPV infection [9, 13–15].

In our study, 1,115 of 4,449 women (25.06%) were identified as HPV-positive, and vaginal infection (BV, VVC, TV, and coinfection) tends to occur in HPV-infected women. BV is the most common type of vaginal infection in adult women. It is characterized by overgrowth of anaerobic bacteria and elevated vaginal pH (>4.5). With the rate of the normal vaginal microecology group served as a control value, the relationship between BV and HPV infection was analyzed, showing a positive correlation with an odds ratio of 1.50. It is possible that *Lactobacillus* producing hydrogen peroxide dominates the vaginal flora as part of the main defense mechanisms in women with normal vaginal microecology, while in patients with BV, the loss of these protective microorganisms along with other changes in the vaginal environment may promote survival of other pathogens, which is a risk factor for developing HPV infection [9, 16]. Another hypothesis is that sialidases are increased in the vaginal secretion of patients with BV, which may disrupt protective mucosal barriers, cause microdamage or alterations of epithelial cells, and increase susceptibility to cervical HPV infection [7, 13].

VVC is one of the most common vaginal infections. Our study showed that the presence of VVC is not associated with an increased risk of HPV infection, which is basically consistent with other studies [15, 17]. It could be speculated that, unlike women with BV, women with *Candida* were likely to have a healthy *Lactobacillus*-predominated vaginal microbiota and, therefore, were not more susceptible to cervical HPV infection. Only a few studies have reported an association between TV and HPV infection, and these findings may be explained by common etiological factors, such as the number of sex partners [18]. Our data (81 women with TV infection enrolled) showed there to be a positive association between HPV infection and TV, and additional studies with more patients enrolled are needed to confirm the conclusion.

Only a few extant studies have analyzed the association between vaginal infections and the types and viral loads of HPV. In our current study, we used a PCR assay, which simultaneously genotype HPV types and quantify the viral loads. About 1,115 women tested positive for HPV, and 795 cases were infected with a single type, accounting for 71.30%. The most common genotypes in the normal vaginal microecology group were HPV58 and HPV53, and in the microecological disorder group, intermediate type BV group, BV group, VVC group, TV group, and coinfection group, the most common genotypes were HPV16 and HPV52. HPV16 and HPV52 appeared to be more common in women suffering from vaginal infection than in women with normal vaginal microecology. Cohort studies have shown a higher risk of developing high-grade squamous intraepithelial lesion (HSIL) in women infected with HPV16 than in those infected with other HR-HPV types [19]. In addition, there may be a positive association between cervical lesions and BV [20]. These findings may suggest that BV infection may be a cofactor and a risk factor of certain types of HPV (such as HPV16) causing cervical lesions. However, further studies are needed to prove this view by expanding the samples with cervical lesion. With the real-time assay, we were also able to observe the viral loads of specific genotypes among groups, and we found no statistically significant differences among groups for HPV16 and HPV52. Our previous research suggested that the correlation between cervical lesions and HPV loads is related to the HPV genotype [12], and the current data suggest that there may be no correlation between vaginal infections and the viral loads of HPV.

Our study is limited by its cross-sectional design, and data on the prevalence of vaginal infection and HPV infection were gathered simultaneously instead of over time, and thus, the data are not suitable for observing the dynamic nature of infections. The relationship of vaginal infections and HPV infection is due to a biological interaction between them, or both occur frequently in a certain group of women, which is yet to be explored. Additional longitudinal and molecular studies are needed.

In summary, our data show that BV and TV are associated with HPV infection, especially high-risk HPV infection, while VVC is not associated with HPV infection. The imbalance of vaginal microecological may be a synergistic risk factor for HPV infection, and further study on the interaction between them can provide new ideas and evidence for the prevention of cervical cancer lesions.

## Figures and Tables

**Table 1:** Distribution characteristics of HPV and vaginal infections in 4,449 women.

	*N* (%)	Negative	HPV positive				
			High-risk	Low-risk	Multiple	Total	Prevalence

Overall	4,449	3,334	741	54	320	1,115	25.06%
Normal vaginal microecology	505 (11.35)	387	81	7	30	118	23.37%
Microecological disorders	1,707 (38.37)	1,292	274	24	117	415	24.31%
Intermediate type BV^a^	1,265 (28.43)	956	197	10	102	309	24.43%
BV^a^	525 (11.80)	360	117	4	44	165	31.43%
VVC^a^	246 (5.53)	197	32	6	11	49	19.92%
TV^a^	81 (1.82)	53	18	2	8	28	34.57%
Coinfection	120 (2.70)	89	22	1	8	31	25.83%

^a^BV, bacterial vaginosis; VVC, vulvovaginal candidiasis; TV, trichomonas vaginalis.

**Table 2:** Odds ratios for prevalence of human papillomavirus among groups.

	OR	95% CI	Wald *χ*^2^	*P* ^b^
Normal vaginal microecology	1.00	—	—	Referent
Microecological disorders	1.05	0.834–1.332	0.196	0.658
Intermediate type BV^a^	1.03	0.910–1.161	0.198	0.657
BV^a^	1.50	1.140–1.982	8.349	0.004
VVC^a^	0.95	0.865–1.044	1.135	0.281
TV^a^	1.12	1.010–1.234	4.603	0.032
Coinfection	1.02	0.947–1.103	0.325	0.569

^a^BV, bacterial vaginosis; VVC, vulvovaginal candidiasis; TV, trichomonas vaginalis. ^b^The odds ratios for prevalence of human papillomavirus among groups were analyzed by the binary logistic regression method.

**Table 3:** HPV types and vaginal infections.

	Overall	Normal vaginal microecology	Microecological disorders	Intermediate type BV^a^	BV^a^	VVC^a^	TV^a^	Coinfection	*P* ^b^
	*N* (%)	*N* (%)	*N* (%)	*N* (%)	*N* (%)	*N* (%)	*N* (%)	*N* (%)	
All	4,449	505	1,707	1,265	525	246	81	120	
HPV positive	1,115 (25.06)	118 (23.37)	415 (24.31)	309 (24.43)	165 (31.43)	49 (19.92)	28 (34.57)	31 (25.83)	*P*=0.002
Single infection	795 (17.87)	88 (17.43)	298 (17.46)	207 (16.36)	121 (23.05)	38 (15.45)	20 (24.69)	23 (19.17)	*P*=0.017
High risk	741 (16.66)	81 (16.04)	274 (16.05)	197 (15.57)	117 (22.29)	32 (13.01)	18 (22.22)	22 (18.33)	*P*=0.006
16+	161 (3.62)	6 (1.19)	57 (3.34)	49 (3.87)	34 (6.48)	7 (2.85)	3 (3.70)	5 (4.17)	
31+	14 (0.31)	0 (0.00)	8 (0.47)	3 (0.24)	3 (0.57)	0 (0.00)	0 (0.00)	0 (0.00)	
33+	33 (0.74)	3 (0.59)	13 (0.76)	12 (0.95)	5 (0.95)	0 (0.00)	0 (0.00)	0 (0.00)	
35+	18 (0.40)	4 (0.79)	8 (0.47)	3 (0.24)	1 (0.19)	0 (0.00)	2 (2.47)	0 (0.00)	
52+	115 (2.58)	8 (1.58)	45 (2.64)	33 (2.61)	17 (3.24)	5 (2.03)	2 (2.47)	5 (4.17)	
58+	76 (1.71)	10 (1.98)	25 (1.46)	21 (1.66)	12 (2.29)	3 (1.22)	2 (2.47)	3 (2.50)	
18+	26 (0.58)	3 (0.59)	8 (0.47)	4 (0.32)	7 (1.33)	2 (0.81)	1 (1.23)	1 (0.83)	
39+	30 (0.67)	6 (1.19)	13 (0.76)	6 (0.47)	1 (0.19)	3 (1.22)	1 (1.23)	0 (0.00)	
45+	10 (0.22)	0 (0.00)	4 (0.23)	5 (0.40)	0 (0.00)	0 (0.00)	1 (1.23)	0 (0.00)	
59+	33 (0.74)	6 (1.19)	12 (0.70)	5 (0.40)	6 (1.14)	1 (0.41)	1 (1.23)	2 (1.67)	
68+	24 (0.54)	5 (0.99)	5 (0.29)	4 (0.32)	7 (1.33)	1 (0.41)	2 (2.47)	0 (0.00)	
53+	68 (1.53)	10 (1.98)	29 (1.70)	17 (1.34)	7 (1.33)	2 (0.81)	1 (1.23)	2 (1.67)	
56+	53 (1.19)	5 (0.99)	19 (1.11)	16 (1.26)	6 (1.14)	3 (1.22)	1 (1.23)	3 (2.50)	
66+	41 (0.92)	9 (1.78)	13 (0.76)	11 (0.87)	6 (1.14)	1 (0.41)	0 (0.00)	1 (0.83)	
51+	39 (0.88)	6 (1.19)	15 (0.88)	8 (0.63)	5 (0.95)	4 (1.63)	1 (1.23)	0 (0.00)	
Low risk	54 (1.21)	7 (1.39)	24 (1.41)	10 (0.79)	4 (0.76)	6 (2.44)	2 (2.47)	1 (0.83)	*P*=0.259
6+	13 (0.29)	0 (0.00)	7 (0.41)	4 (0.32)	0 (0.00)	2 (0.81)	0 (0.00)	0 (0.00)	
11+	7 (0.16)	1 (0.20)	3 (0.18)	0 (0.00)	1 (0.19)	1 (0.41)	1 (1.23)	0 (0.00)	
81+	34 (0.76)	6 (1.19)	14 (0.82)	6 (0.47)	3 (0.57)	3 (1.22)	1 (1.23)	1 (0.83)	
Multiple infection	320 (7.19)	30 (5.94)	117 (6.85)	102 (8.06)	44 (8.38)	11 (4.47)	8 (9.88)	8 (6.67)	*P*=0.623

^a^BV, bacterial vaginosis; VVC, vulvovaginal candidiasis; TV, trichomonas vaginalis. ^b^The prevalence of HPV genotypes among different groups was analyzed by *χ*^2^ test.

## Data Availability

The data used to support the findings of this study are available from the corresponding author upon request.
